# Identifying Determinants of Cullin Binding Specificity Among the Three Functionally Different *Drosophila melanogaster* Roc Proteins via Domain Swapping

**DOI:** 10.1371/journal.pone.0002918

**Published:** 2008-08-13

**Authors:** Patrick J. Reynolds, Jeffrey R. Simms, Robert J. Duronio

**Affiliations:** 1 Department of Biology, University of North Carolina, Chapel Hill, North Carolina, United States of America; 2 Program in Molecular Biology and Biotechnology, University of North Carolina, Chapel Hill, North Carolina, United States of America; 3 Lineberger Comprehensive Cancer Center, University of North Carolina, Chapel Hill, North Carolina, United States of America; Institut Pasteur, France

## Abstract

**Background:**

Cullin-dependent E3 ubiquitin ligases (CDL) are key regulators of protein destruction that participate in a wide range of cell biological processes. The Roc subunit of CDL contains an evolutionarily conserved RING domain that binds ubiquitin charged E2 and is essential for ubiquitylation. *Drosophila melanogaster* contains three highly related Roc proteins: Roc1a and Roc2, which are conserved in vertebrates, and Roc1b, which is specific to *Drosophila*. Our previous genetic data analyzing *Roc1a* and *Roc1b* mutants suggested that Roc proteins are functionally distinct, but the molecular basis for this distinction is not known.

**Methodology/Principal Findings:**

Using co-immunoprecipitation studies we show that *Drosophila* Roc proteins bind specific Cullins: Roc1a binds Cul1-4, Roc1b binds Cul3, and Roc2 binds Cul5. Through domain swapping experiments, we demonstrate that Cullin binding specificity is strongly influenced by the Roc NH_2_-terminal domain, which forms an inter-molecular β sheet with the Cullin. Substitution of the Roc1a RING domain with that of Roc1b results in a protein with similar Cullin binding properties to Roc1a that is active as an E3 ligase but cannot complement *Roc1a* mutant lethality, indicating that the identity of the RING domain can be an important determinant of CDL function. In contrast, the converse chimeric protein with a substitution of the Roc1b RING domain with that of Roc1a can rescue the male sterility of *Roc1b* mutants, but only when expressed from the endogenous *Roc1b* promoter. We also identified mutations of *Roc2* and *Cul5* and show that they cause no overt developmental phenotype, consistent with our finding that Roc2 and Cul5 proteins are exclusive binding partners, which others have observed in human cells as well.

**Conclusions:**

The *Drosophila* Roc proteins are highly similar, but have diverged during evolution to bind a distinct set of Cullins and to utilize RING domains that have overlapping, but not identical, function *in vivo*.

## Introduction

Many aspects of cell and developmental biology require the regulation of protein function via ubiquitin-mediated protein destruction. Protein ubiquitylation requires the action of three families of proteins: E1 Ubiquitin Activators (Uba), E2 Ubiquitin Conjugators (Ubc), and E3 Ubiquitin Ligases (Ubl) [1], [2]. Ubiquitin monomers are activated through conjugation via a thiolester linkage to an internal cysteine in E1, which then transfers the ubiquitin to a cysteine residue of an E2 protein. The E2 interacts with an E3 to mediate covalent attachment of ubiquitin onto substrate proteins. Repeated rounds of E2/E3-mediated ubiquitin transfer result in polyubiquitylation, allowing substrate proteins to be recognized and destroyed by the proteasome. Vertebrates and sea urchins have two distinct E1 enzymes, and most other organisms are thought to only have a single E1 [3], [4]. While there are considerably more E2's, much of the modularity of the ubiquitin-proteasome pathway comes from the large number of different E3 ligases.

E3's can be broadly categorized as either HECT domain or RING domain. Ubiquitin is directly conjugated to an internal cysteine residue of HECT domain E3's before being transferred onto the substrate protein [5]. RING E3's do not conjugate ubiquitin, but rather stimulate its transfer from the E2 to the substrate [6], [7]. RING domains contain conserved cysteine and histidine residues that chelate zinc ions to provide a structure that interacts with an E2 [8], [9]. Several RING domain structures have been solved, and they share extensive structural conservation [10], [11], [12], [13], [14]. At least two of these RING proteins, c-Cbl and Rbx1/Roc1, use a similar hydrophobic groove in the protein to bind an E2. All of the Zn^++^ chelating residues of the RING domain (as well as the Zn^++^ ions) are required for proper folding and function of the protein [7], [9], [15], [16], [17]. The importance to E3 ligase function of residues or domains outside of the RING domain is not currently understood.

RING E3's can be further categorized as either single or multi-protein complexes. Single protein E3's, like c-Cbl, perform the entire function of the E3 within the context of one polypeptide [18]. Multi-protein RING E3 ligases, like the Anaphase Promoting Complex (APC) and Cullin-Dependent Ligases (CDL), use a complex of many different proteins to facilitate ubiquitin transfer [18]. CDL are composed of three modules (Cullin, Roc, substrate adapter/receptor), each with a distinct role [19]. The Cullin serves as a scaffold, binding a Roc protein at its COOH-terminus and a substrate adapter/receptor module at its NH_2_-terminus. The Roc protein serves as an interaction surface for ubiquitin-bound E2, and thereby recruits charged ubiquitin to the E3 ligase machinery. The substrate adapter/receptor module (a single protein in Cul3 CDL and multiple proteins in all other CDL) binds directly and specifically to one or a small subset of proteins targeted for polyubiquitylation and destruction. Large gene families encode these substrate adapter/receptor modules, which are thought to provide most of the CDL substrate specificity [20], [21]. For instance, the F-box family (33 members in *Drosophila* [22]) contains a diverse group of proteins that recruit specific substrates to the Cul1 E3 ligase via the Skp1 adaptor, which binds both the NH_2_-terminus of Cul1 and the F-box domain [11], [23].

The RING domain-containing Roc proteins are essential for CDL function and are also encoded by a gene family [19]. Humans and *C. elegans* contain two Roc proteins, Roc1 and Roc2, while there has been a radiation of the Roc1 family in *Drosophilid* species (T.D. Donaldson and R.J.D., unpublished). For instance, *Drosophila melanogaster* encodes three Roc proteins named *Roc1a*, *Roc1b* and *Roc2* [24]. The level of functional redundancy among metazoan Roc proteins has remained largely unexplored. We have been addressing this issue in *Drosophila melanogaster* by generating and characterizing mutations in the *Roc* genes. We previously showed that *Roc1a* mutants are lethal, while *Roc1b* mutants are male sterile [24], [25]. These different phenotypes suggest that the Rocs are not redundant in function, even though Roc1a and Roc1b are 78% identical in the RING domain. Moreover, this lack of redundancy is not a result of tissue specific expression, since full compensation of *Roc1a* mutant phenotypes in wing imaginal cells cannot be achieved via over-expression of either Roc1b or Roc2 [25]. These data suggest that there exist intrinsic differences in the highly related Roc proteins. Here we show that each *Drosophila* Roc protein binds a distinct set of Cullin proteins. We analyze chimeras between the three Roc proteins to map binding determinants, and demonstrate that both the RING and NH_2_-terminus of the Roc proteins can influence Cullin binding. Further, we show that not all RING domains of the individual Roc proteins are functionally interchangeable *in vivo*. This suggests that rather than simply providing an E2 binding interface for Cullin proteins, the Roc proteins are structurally distinct and specific RING domains play an important role in determining overall CDL function during development.

## Results

### Roc proteins bind specific Cullins

**Figure 1:**
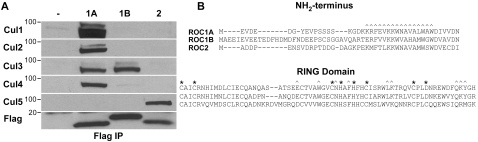
Roc-Cullin interactions in vivo. *A*, Flag-Roc protein complexes were immunoprecipitated from extracts prepared from wild type (−), Flag-Roc1a, Flag-Roc1b, and Flag-Roc2 transgenic embryos, and co-precipitating Cullin proteins were detected by immunoblotting. *B*, Alignment of *Drosophila* Roc proteins, indicating how we designated NH_2_-terminal and RING domains. Asterisks indicate the Zn^++^ chelating residues of the RING domain that are essential for ligase function, and the carets indicate amino acids that make important contacts with Cullins. These assignments are based on the Cul1-Rbx1 structure of Zheng et al [11].

We previously detected Roc-Cullin interactions *in vivo* by immunoprecipitating Flag-tagged Roc1a, Roc1b, and Roc2 proteins from transgenic embryo extracts and identifying interacting proteins by mass spectrometry [25]. For these experiments, each Roc protein was expressed using the ubiquitous *Roc1a* promoter [24]. The data indicated that Roc1a bound Cul1-4 and that Roc2 bound Cul5, but could not eliminate the possibility that certain Roc-Cullin complexes were undetectable by the IP/mass spec analysis. To more comprehensively test for the presence of Roc-Cullin complexes, we assembled a panel of anti-Cullin antibodies (see methods) and used these in IP/immunoblot analyses. Anti-FLAG immunoprecipitates of extracts made from transgenic embryos expressing FLAG-tagged Roc1a, Roc1b, or Roc2 proteins from the *Roc1a* promoter were probed with antibodies that specifically recognize each Cullin (Figure 1A). The data indicate that Roc1a binds Cullins 1–4, but not Cul5, that Roc1b binds strongly only to Cul3, and that Roc2 binds only to Cul5. This is consistent with our previous mass spec results [25] and shows that Rocs have strong Cullin binding preferences *in vivo*.

### The Roc Protein NH_2_-terminus can determine Cullin binding specificity

**Figure 2:**
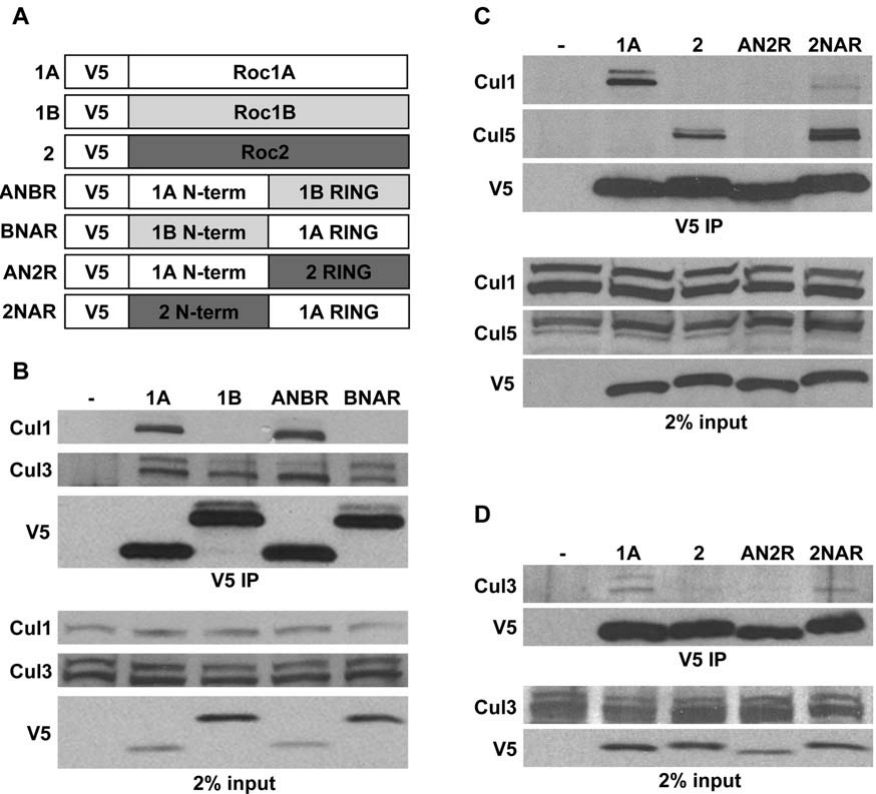
RING swap constructs and Cullin binding in S2 cells. *A*, Schematic representation of the RING swap constructs. All constructs are expressed with the *Roc1a* promoter and regulatory regions, and the chimeric proteins contain an amino-terminal V5 tag. *B*, Control Roc1a and Roc1b or ANBR and BNAR constructs were transfected into S2 cells and cellular extracts were immunoprecipitated with anti-V5 antibodies and probed for the presence of Cul1 and Cul3 by immunoblotting. *C*, Control Roc1a and Roc2 or AN2R and 2NAR constructs were transfected into S2 cells and cellular extracts were immunoprecipitated with anti-V5 antibodies and probed for the presence of Cul1 and Cul5 by immunoblotting. *D*, AN2R and 2NAR chimeras were tested for interaction with Cul3 as in B, C.

Available crystal structures of Roc-Cullin complexes (e.g. Roc1-Cul1 and Roc1-Cul4) reveal that the ∼110 amino acid Roc proteins contain two general domains, an NH_2_-terminal β-strand of between 41 and 56 amino acids and a globular RING domain that chelates 3 zinc ions (Fig. 1B) [11], [14]. Deletion of the NH_2_-terminal β-strand of human Roc1 abrogates binding to Cul1 [26], indicating that this domain is required for Cullin binding. Both the NH_2_-terminal β-strand and the RING domain make close contacts with the Cullin homology domain at the Cullin COOH-terminus, and thus could potentially mediate specific Cullin-Roc interaction (Fig. 1B) [11]. However, because the sequence of the RING domain is more highly conserved than the NH_2_-terminal β-strand, we hypothesized that the NH_2_-terminus of Roc proteins is responsible for mediating *specific* Cullin interactions. For instance, the Roc1a and Roc1b RING domains are 78% identical (Fig. 1B), yet Roc1b binds strongly only to Cul3 while Roc1a binds strongly to Cullins 1–4 (Fig. 1A). To determine whether one of these two domains in Roc proteins is responsible for specific Cullin binding, we created a series of chimeric constructs that join the NH_2_-terminus of one Roc protein to the COOH-terminal RING domain of another (RING-swap constructs, Fig. 2A). For example, AN2R contains the Roc1a NH_2_-terminus fused to the Roc2 RING domain. All constructs have an NH_2_-terminal V5 epitope tag and are expressed with the *Roc1a* promoter. If the NH_2_-terminus is sufficient to confer Cullin binding specificity, all chimeras with the same NH_2_-terminal Roc sequence should bind the same Cullins, regardless of the RING domain to which they are fused.

We transfected these constructs into S2 cells and determined Cullin binding by IP/immunoblot. Because we had already observed that Roc1a binds Cul1 and Cul3 but not Cul5, that Roc1b binds only Cul3, and that Roc2 binds only Cul5 (Fig. 1A), we focused our analysis on these interactions. The ANBR chimera showed the same binding preference as Roc1a: it bound both Cul1 and Cul3 (Fig. 2B). Similarly, the BNAR chimera displayed the same binding specificity as Roc1b: it bound Cul3 but not Cul1 (Fig. 2B). These data indicate that the NH_2_-terminus of a Roc protein can direct specific Cullin binding.

Chimeras between Roc1a and Roc2 behaved slightly differently than the ANBR and BNAR proteins. For instance, AN2R failed to bind any Cullin, even though it could be stably expressed (shown for Cul1 and Cul5 in Fig. 2C). There could be several reasons why AN2R fails to bind Cullin. There may be amino acids in the Roc1a RING domain necessary for Cul1 binding that are not present in the Roc2 RING domain. We swapped several potential specificity residues in the RING domain between Roc1a and Roc2, but were unable to alter the Cullin binding (data not shown). Alternatively, since ANBR binds to Cul1, the Roc2 RING domain may contain amino acids that prevent binding to Cul1. However, we show below that the AN2R protein is not active as an E3 ligase (for example, see Fig. 6 below), and thus this protein chimera is functionally inactive perhaps because it does not fold properly. In the reciprocal experiment, 2NAR bound strongly to Cul5, and more weakly to Cul1 (Fig. 2C). Interestingly, we could also detect some binding of 2NAR to Cul3 (Figure 2D). These data indicate that the Roc2 NH_2_-terminus confers strong binding preference to Cul5, and that the Roc1a RING domain contributes somewhat to the selection of Cul1 and Cul3.

**Figure 3:**
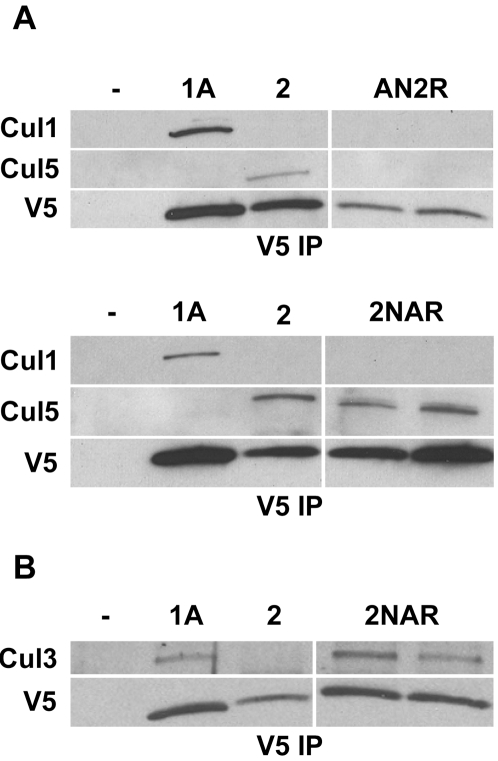
RING Swap constructs and Cullin binding in embryos. *A*, RING-swap chimeric proteins were immunoprecipitated from transgenic embryo extracts and tested for interaction with Cul1 and Cul5 by immunoblotting. Two independent transgenic lines of AN2R and 2NAR are shown. *B*, Two 2NAR transgenic lines were tested for interaction with Cul3.

To confirm some of these observations *in vivo*, we generated multiple AN2R and 2NAR transgenic lines and analyzed embryo extracts by IP-Western analysis as in Figure 1. We consistently obtained similar results as in S2 cells; AN2R bound no Cullin, while 2NAR bound Cul5 but not Cul1 as did normal Roc2 (Fig. 3A). In addition, 2NAR also bound Cul3 (Fig. 3B). Thus, the Roc NH_2_-terminus provides a strong determinant for Cullin binding specificity, but is not always sufficient. In addition, since full length Roc2 does not bind either Cul1 or Cul3, our data suggest that the RING domain of Roc1a plays a more important role in Cullin binding specificity than the RING domain of Roc2.

Nedd8 is a small ubiquitin-like protein that is conjugated to the K720 residue of Cul-1 (and to an homologous lysine in all other Cullins as well) using Uba3 (E1), Ubc12 (E2), and Roc1 (E3) [28], [29], [30], [31]. This modification is in turn cleaved off by the multi-subunit COP9 Signalosome (CSN) [32], [33]. Since mutations in the pathways that conjugate and remove Nedd8 are detrimental, the current model is that cycles of neddylation and de-neddylation are necessary for the proper function of CDL [34], [35], [36]. We observed both the unmodified and the less abundant, slower migrating neddylated form of Cullin in some of our experiments (e.g. Fig. 1 Cullins 1–3). Because Roc1 can influence Cullin neddylation, we examined our co-immunoprecipitation data paying particular attention to whether association with different Roc chimeras affects the steady state amount of Cullin neddylation. We could find no consistent correlation between Cullin neddylation and a particular domain of one of the Roc proteins.

### The Roc1b RING domain cannot provide all Roc1a function

**Figure 4:**
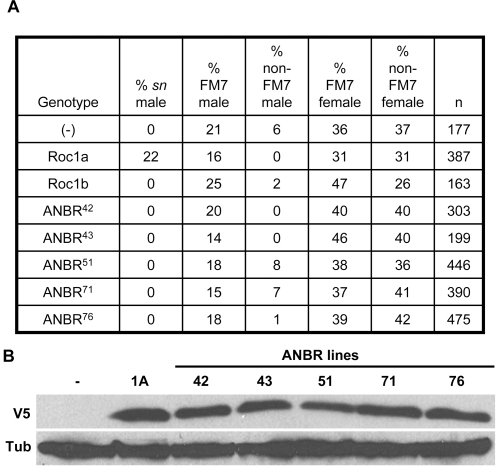
ANBR does not rescue the lethality of *Roc1a* mutation. *A*, Transgenes expressing chimeric ANBR proteins were tested for rescue of *Roc1a^G1^* lethality (see *Methods* for genetics). All F1 progeny contain a single copy of the transgene. The transgenes are expressed under control of the *Roc1a* regulatory sequences. The percentage of progeny with each genotype is indicated, as is the total number of progeny scored (n). *B*, V5 immunoblot of extracts prepared from adult males containing the indicated transgenes. (−) indicates non-transgenic wild type control.

Since the ANBR protein displays the same binding specificity as Roc1a, we wanted to determine whether it could rescue the lethality caused by the null *Roc1a^G1^* mutation [24]. *Roc1a* is located on the X chromosome, and thus *Roc1a^G1^* males are not viable. We set up an experiment where fathers carrying an autosomally located Roc1a (as control) or ANBR transgene (expressed with the *Roc1a* promoter) were crossed to *Roc1a^G1^*/*FM7* mothers and scored for the presence of rescued *Roc1a^G1^* males. While the wild type *Roc1a* transgene was able to rescue the lethality of the *Roc1a^G1^* mutation, none of 5 different ANBR transgenic insertions were able to do so (Fig. 4A). This was not a result of expression level differences, because all 5 of the ANBR proteins accumulated to a level comparable to the control Roc1a transgenic protein (Fig. 4B). Thus, while ANBR binds Cul1 (Fig. 2) and Cul2-4 (not shown) similarly to Roc1a, it is unable to function the same as Roc1a. This was somewhat surprising, considering that Roc1a and Roc1b share 78% protein identity in the RING domain. One possibility is that the Roc1b RING domain is unable to interact with the same E2's as the Roc1a RING domain when assembled into Cullin complexes, which would suggest that at least one essential target of a Roc1a E3 ligase is dependent on the specific RING domain sequence of Roc1a. The AN2R construct was also unable to rescue *Roc1a^G1^* lethality (data not shown), consistent with the failure of the AN2R protein to bind Cullin.

In several of these rescue experiment crosses we detected a small number of unbalanced male progeny that did not display the *sn* phenotype, and thus that did not contain the *Roc1a^G1^* mutant chromosome (Fig. 4A). We do not unambiguously know the origin of these males, but since they were invariably sterile they are likely XO male progeny resulting from meiotic non-disjunction events in the *Roc1a^G1^*/FM7 females. This raises the possibility that reduction of *Roc1a* gene dose in females affects meiotic chromosome segregation.

### Chimeric Roc proteins can function in vivo

**Figure 5:**
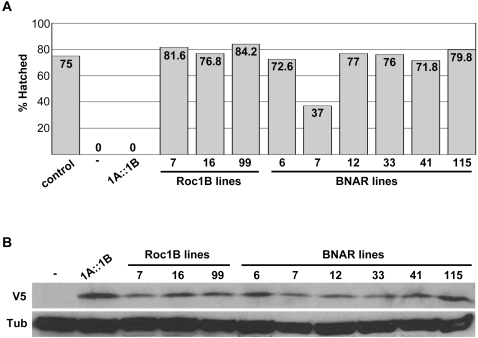
BNAR rescues the *Roc1b* mutant male sterility. *A*, Egg hatching was assessed for progeny of *Roc1b^dc3^* homozygous mutant males containing the indicated transgene. “Control” indicates *Roc1b^dc3^/+* genotype. (−) indicates no transgene; i.e. a *Roc1b^dc3^* homozygous mutant. *1A::1B* indicates Roc1b driven by the *Roc1a* regulatory sequences. All other lines are under control of the *Roc1b* regulatory sequences. 500 eggs were analyzed for each line. *B*, V5 immunoblot of extracts prepared from adult males containing the indicated transgenes.

Recently, Arama et al. showed that a testes-specific Cul3 isoform forms an E3 ligase with Roc1b in the testes, and *Roc1b* mutant males are sterile because of a failure to complete the late stages of sperm differentiation [37]. Since the BNAR construct displays the same binding specificity as Roc1b, we tested whether the BNAR chimera could rescue the male sterility caused by the *Roc1b^dc3^* null mutation (Fig. 5)[25]. Male fertility was measured by determining the proportion of eggs that hatched into first instar larvae after mating to wild type females. Three different transgenic lines expressing V5 epitope-tagged Roc1b under the control of the *Roc1b* promoter were able to rescue the male sterile phenotype (Fig. 5A). Six different BNAR lines rescued the male sterility defect, five of them to the level of the control Rob1b transgenes (Fig. 5A). The BNAR chimeric proteins were expressed from the *Roc1b* promoter at levels comparable to normal Roc1b (Figure 5b). Because BNAR binds to Cul3, we conclude that the Roc1a RING domain can provide Roc1b function during spermatogenesis. This is consistent with our previous observations indicating the forced expression of normal Roc1a from the *Roc1b* promoter can partially rescue the *Roc1b* male fertility defect [25]. When considered together with the failure of ANBR to rescue the *Roc1a* mutant, these results suggest that, within the context of the male germ line-specific Cul3 E3 ligase complex, the Roc1a RING domain can productively interact with the same E2s as Roc1b, while Roc1b cannot do so with all of the E2s that function with Roc1a.

Interestingly, expression of wild type Roc1b from the *Roc1a* promoter was unable to rescue male sterility (Fig. 5A). This suggests that while the *Roc1a* promoter is active in the male body [24], it is not expressed in the male germ line in a manner appropriate to provide Roc1b function. Thus, the *Roc1b* regulatory sequence appears to confer expression in the testes that cannot be duplicated by the *Roc1a* promoter.

### Chimeric Roc proteins have E3 ligase activity in vitro

**Figure 6:**
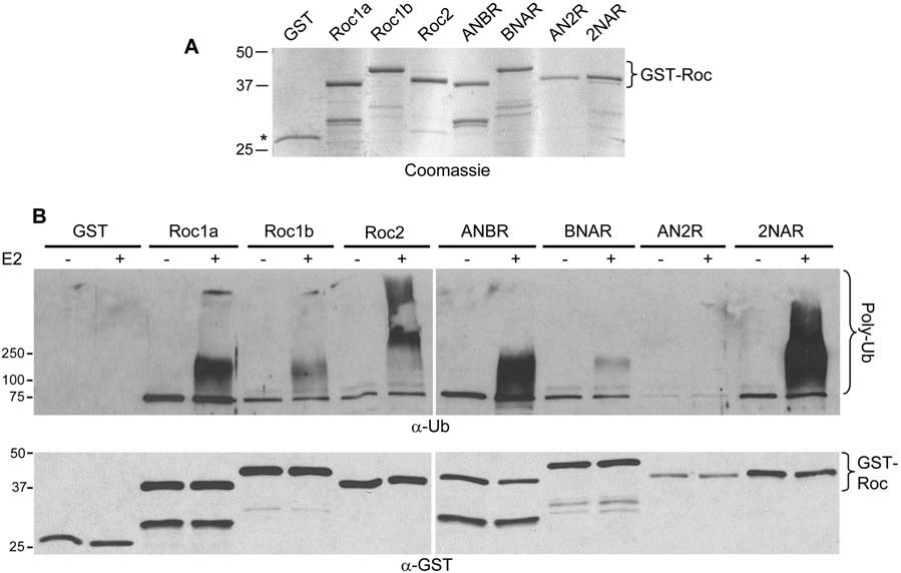
Ligase activity of RING swap proteins. *A*, Coomassie stained gel of purified GST-Roc proteins used in the ligase assays. Bracket indicates GST-Roc proteins. Asterisk indicates GST. Lower bands are degradation products. *B*, All *Drosophila* Roc proteins and chimeras were assessed for ligase activity in a substrate free assay. Briefly, 250 ng of Roc protein (or control GST) were added to a ubiquitin ligase mixture containing UbcH5, ubiquitin, and ligase buffer and incubated for 45 minutes. “−” and “+” indicate the absence or presence of UbcH5 in the reaction, respectively. Ubiquitin conjugates were detected by Western using an anti-Ub antibody (bracket indicates poly-ubiquitin chains), and GST-Roc proteins were detected using an anti-GST antibody.

Although ANBR binds Cul1-4, it does not rescue the lethality of *Roc1a* mutation. A possible explanation for this result is that the ANBR chimera protein is deficient in E3 ligase activity. To test this, we expressed all of the *Drosophila* Roc proteins and chimeras as GST fusion proteins in *E. coli* and purified them (Fig. 6A). We then tested them for E3 ligase activity using a previously described in vitro assay that detects E2- and GST-Roc-dependent polyubiquitin formation in the absence of either Cullin or a particular substrate [24], [26]. The ANBR protein was fully functional in this assay (Fig. 6B). Thus, ANBR can bind Cullin and function as an E3 ligase, but it cannot provide all the function of Roc1a *in vivo*.

Roc1a and Roc2 displayed high E3 ligase activity, whereas Roc1b showed a weaker ability to promote poly-ubiquitylation (Fig. 6B). Comparing this activity with that of the chimeras, a pattern emerges: Roc1a and ANBR promote extensive polyubiquitylation, while that of Roc1b and BNAR is lower. Similarly, Roc2 and the 2NAR chimera are both highly active in this assay (Fig. 6B). Thus, even though the RING domain is known to mediate most of the physical interaction with the E2 [11], the Roc NH_2_-terminus may play a significant role in determining the efficiency of poly-ubiquitylation with a particular E2. As noted above, in addition to its inability to bind Cullin (Fig. 2C & 2D), the AN2R protein is non-functional as an E3 ligase in this assay (Fig. 6B).

### Roc2 and Cul5 Mutant Analysis

**Figure 7:**
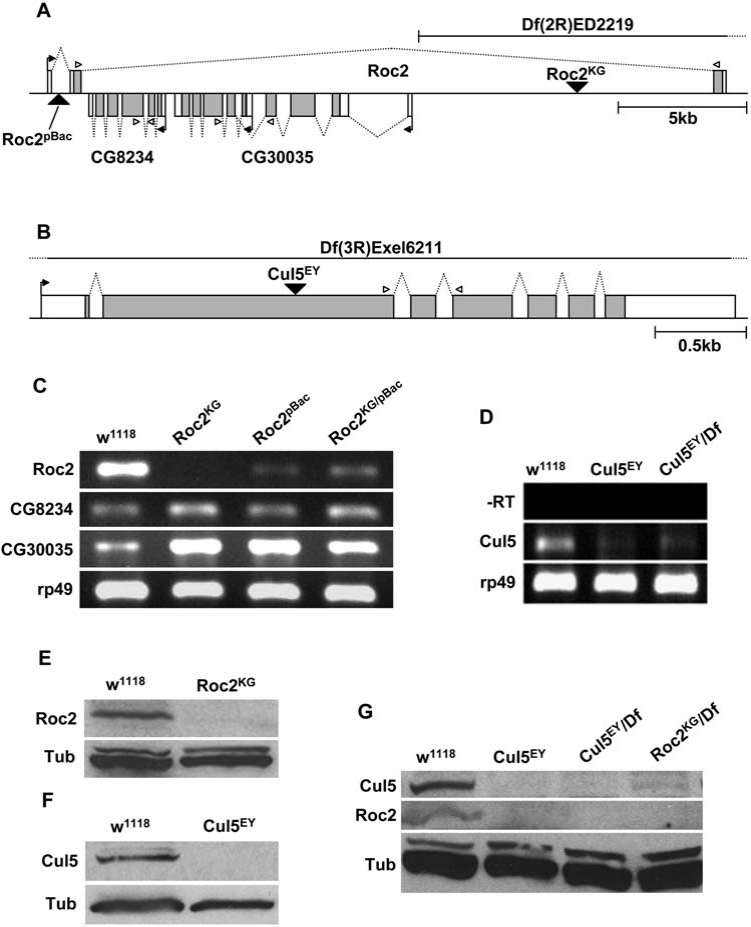
Analysis of *Roc2* and *Cul5* mutant alleles. *A*, Schematic of the *Roc2* locus. CG8234 and CG30035 are genes of unknown function as annotated by FlyBase (putative sugar transporters). *B*, Schematic of the *Cul5* locus. Right angle arrows indicate start of transcription. Open arrow heads show the position of primers used for RT-PCR. Larger black triangles are P-element or piggyBac insertions. The boxes indicate exons, and the shaded regions represent the open reading frame. Dotted line indicates splicing. *C*, RT-PCR analysis of the *Roc2* alleles. KG and pBac are homozygous for the insertions, and KG/pBac is a transheterozygote. Ribosomal protein 49 (rp49) was used as a positive control. *D*, RT-PCR analysis of the Cul5 allele. −RT indicates that no reverse transcriptase was added. *E*, Immunoblot comparing Roc2 protein levels in wild-type (*w^1118^*) and homozygous *Roc2^KG^* embryos. *F*, Immunoblot comparing Cul5 protein levels in wild-type and homozygous Cul5^EY^ embryos. In each case the embryos were derived from crosses between mutant mothers and fathers. *G*, Embryo extracts from *Cul5* and *Roc2* mutants were blotted with antibodies against the respective proteins.

Because Roc2 and Cul5 bind only to each other, we hypothesized that a Roc2-Cul5 complex would function independently of other Roc-Cullin complexes and that mutations of each gene would cause the same phenotype. In addition, this hypothesis predicts that the *Roc2* mutant phenotype would be different than the phenotype we previously determined for *Roc1a* and *Roc1b* mutants [24], [25]. The *Roc2* locus is complex, with two protein coding exons separated by an intron greater than 25 kb in length (Fig. 7A). Within this intron are two predicted genes (CG8234 or CG30035) that encode sugar transporters transcribed from the strand opposite *Roc2* transcription (Fig. 7A). We analyzed several transposon insertion lines for expression of Roc2 and identified two mutant alleles, both of which are viable. The *Roc2^KG^* P element insertion is within the large intron, while the *Roc2^pBac^* piggyBac insertion is in a smaller intron upstream of the first protein-coding exon (Fig. 7A). RT-PCR analysis of embryonic mRNA (from mating homozygous males and virgin females) revealed that the *Roc2^KG^* allele expresses no detectable mRNA, while the *Roc2^pBac^* allele expresses a reduced amount of mRNA compared to wild type (Fig. 7C). Neither insertion reduces the expression of the CG8234 or CG30035 genes (Fig. 7C). The *Roc2^KG^* allele also produces no detectable Roc2 protein as measure by immunoblotting of embryonic protein extracts (Fig. 7E), and thus it is a strong loss of function mutation. *Roc2^KG^* mutant animals develop normally, and we could detect no obvious phenotype, except for a slight reduction in female fecundity.

The *Cul5* locus is simpler than that of *Roc2*, and the *Cul5* transcript includes 7 exons and 6 introns. We identified a P element (*Cul5^EY^*) that is inserted into the second exon of *Cul5* at amino acid D346 of the predicted open reading frame (Fig. 7B). RT-PCR analysis of embryonic mRNA (from homozygous mutant males and virgin females) revealed a reduced level of Cul5 mRNA in *Cul5^EY^* (Fig. 7D), and we were unable to detect Cul5 protein by immunoblotting of protein extracts from the same mating (Fig. 7F), indicating that *Cul5^EY^* is a strong loss of function allele. As with *Roc2*, *Cul5^EY^* mutants develop normally and do not display any overt morphological defects. That *Roc2* and *Cul5* mutant animals are viable and display no obvious developmental defects is consistent with our analysis of Roc-Cullin interactions indicating that Roc2 and Cul5 bind exclusively to each other. Indeed, Roc2 and Cul5 accumulation *in vivo* is interdependent: Roc2 was undetectable in *Cul5* mutant embryo extracts, and Cul5 was greatly reduced in *Roc2* mutant embryo extracts (Fig. 7G).

**Figure 8:**
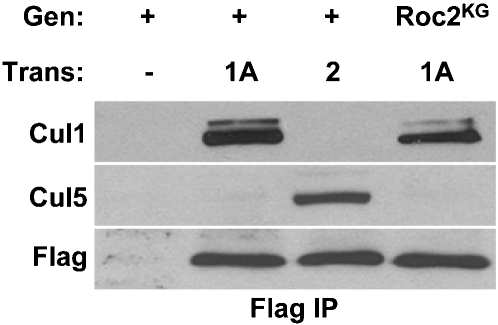
Roc2 and Cul5 are exclusive binding partners. *A*, Extracts were prepared from embryos with either a wild-type (Gen = +) or *Roc2* mutant (Gen = *Roc2^KG^*) background and the indicated transgene. The immunoprecipitated FLAG-Roc protein is indicated at the top. (−) indicates no transgene.

While Roc1a does not bind Cul5 when Roc2 protein is present, it is possible that we were unable to observe a phenotype in *Roc2* mutants because Roc1a can bind Cul5 in the absence of Roc2, and thereby functionally substitute for Roc2. We tested this by introducing a *Roc1a* transgene into the *Roc2^KG^* mutant background. Even in this genotype, Roc1a bound to Cul1 but not detectably to Cul5 (Fig. 8). Moreover, as we show in Fig. 6G, the pool of Cul5 available for binding Roc1a is greatly reduced in *Roc2* mutant animals relative to wild type. Thus, the Cul1 and Cul5 E3 ligase complexes form independently of one another and do not compete for the same pool of Roc proteins.

## Discussion

In this study we show that the Roc proteins play a part in the functional modularity of Cullin-dependent E3 ligases. Our data show that selective Roc-Cullin interactions occur *in vivo* in *D. melanogaster*, and that the highly conserved Roc proteins serve distinct roles as members of different Cullin E3 ligase complexes.

### Roc-Cullin interaction determinants

The Roc NH_2_-terminus is necessary for binding to Cullin protein [26] and forms a β-strand that makes an inter-molecular β-sheet with the Cullin protein [11], [14]. Our analysis of “RING swap” protein chimeras indicates that in some instances the Roc NH_2_-terminal β-strand is the primary contributor to Roc-Cullin binding *specificity*. Both fusing the Roc2 NH_2_ terminus to the Roc1a RING domain (2NAR) and fusing the Roc1a NH_2_-terminus to Rob1b (ANBR) results in proteins that primarily display the Cullin binding preferences of Roc2 and Roc1a, respectively. However, our data clearly indicate that the RING domain also makes a contribution to Cullin binding preference. While a full length Roc1a construct bound Cul1-4 (Fig. 1a), a construct consisting of the Roc1a NH_2_ terminus fused to the RING domain of the more distantly related Roc2 (AN2R) was unable to bind any Cullin. Substituting in the Roc1b RING domain, however, was able to restore Cullin binding, and the resulting ANBR protein displayed a Cullin binding profile identical to Roc1a. This suggests that a region of the RING domain that is more similar between Roc1a and Roc1b than Roc2 participates in Roc1a-Cullin binding preferences. Roc1a is 78% identical to Roc1b across the RING domain, while it only shares 45% identity with Roc2 in this region. In addition, the 2NAR protein bound detectably to Cul3 while normal Roc2 protein did not, again suggesting that the Roc1a RING domain influences Cullin selection. Taken together, we conclude from our data that the Roc NH_2_-terminal β-strand makes a relatively stronger contribution to Cullin binding preference than the RING domain.

The lethality of *Roc1a* mutants is presumably caused by the inappropriate accumulation of at least one target of a Roc1a-containing Cullin dependent E3 ligase [24]. The ANBR protein cannot rescue the lethality of *Roc1a* mutants even though it binds all of the Cullins that Roc1a does. Thus, even though ANBR can activate polyubiquitin formation *in vitro*, fusion of the Roc1a NH_2_ terminus with the Roc1b RING domain does not create a fully biologically active Roc1a protein. One possible interpretation of this result is that *in vivo* the Roc1b RING domain cannot productively interact (i.e. stimulate ubiquitylation of critical targets) with all of the E2s that Roc1a does. This may occur at the level of direct binding, such that there are some E2s that only bind the RING domain of Roc1a and not Roc1b, or at the level of stimulating ubiquitin transfer to substrate in the context of a fully assembled CDL.

A similar observation was previously reported for TRAF3 and TRAF5 RING domain proteins [38]. TRAF5 can activate the NF-κB pathway when over-expressed, while TRAF3 cannot. This is dependent on the RING domain, as mutation of a key RING cysteine inhibits this activation. A construct that fuses portions of the RING domains of TRAF3 and TRAF5 is also incapable of NF-κB activation. The authors proposed that these chimeric proteins might not be able to chelate zinc ions or fold properly. This may explain why AN2R is unable to bind any Cullin. However, the other Roc chimeras we constructed were able to bind Cullin and were active as E3 ligases. Moreover, the rescue of male sterility by the ANBR chimera (see below) indicates that Roc protein chimeras *can* be functional *in vivo*.

In the *Drosophila* male germ line, Roc1b forms a complex with a testes-specific Cul3 isoform and the BTB protein KLH10 to regulate caspase activation during spermatid differentiation [37]. Consequently, *Roc1b* mutants are male sterile [25]. Interestingly, the BNAR chimera, which binds well to Cul3, effectively rescues *Roc1b* mutant male sterility. This is consistent with our previous data showing that normal Roc1a can partially rescue the male sterility of *Roc1b* mutants when expressed from the *Roc1b* promoter [25]. These data suggest that in the context of the testes specific Cul3 complex, the RING domains of Roc1a and Roc1b can productively interact with a similar set of E2s. Thus, our genetic rescue experiments with *Roc1a* and *Roc1b* mutants can be explained if Roc1b interacts with a subset of all the E2s that interact with Roc1a. Finally, the functional redundancy of Roc1a and Roc1b in the male germ line is only detected with the *Roc1b* promoter, suggesting that the *Roc1a* and *Roc1b* genes are expressed differently during spermatogenesis.

### Function of the Roc2-Cul5 E3 ligase

We show that Roc2 and Cul5 only bind to each other, and that knocking out one protein greatly reduces the level of the other. The Roc2-Cul5 complex is conserved in other species including *C. elegans* and humans [39], [40]. What is the function of the Roc2-Cul5 complex *in vivo*? A previous *Drosophila* study used viable P element insertions in the 5′UTR of Cul5 for over-expression experiments that suggested Cul5 is involved in cell fate specification and bouton formation in the larval CNS [41]. This study indicated that the insertion alleles were very weakly hypomorphic, and consistent with this we were not able to detect a difference in the Cul5 mRNA levels of these alleles by RT-PCR (data not shown). Here we report the identification of *Roc2* and *Cul5* transposon insertion alleles in which we cannot detect protein by immunoblot analysis of mutant embryos. These mutants develop into morphologically normal adults. Thus, Roc2-Cul5 is not required for development.

It is possible that Roc2-Cul5 is redundant with other CDL. A recent paper showed that Roc1-Cul2 and Roc2-Cul5 complexes may act redundantly during meiotic cell cycle progression in *C. elegans* [39]. RNAi knockdown of Roc2 or Cul5 did not reveal an obvious phenotype, consistent with our results. However, RNAi knockdown of either Roc2 or Cul5 mRNA in a *cul-2* mutant background caused complete sterility, whereas *cul-2* mutants only display partial sterility. We occasionally observed a small, but inconsistent reduction in female fecundity in both the *Drosophila Roc2* and *Cul5* mutants, perhaps reflecting such redundancy. Cul2- and Cul5-based E3 ligases use similar substrate adapter machinery, consisting of ElonginB, ElonginC, and a variable BC box protein [40], [42], [43], suggesting that Cul2 and Cul5 complexes could have overlapping substrates in some organisms. *Drosophila* Cul2 forms a complex with Rbx1, Elongins B and C, and VHL that supports polyubiquitin chain formation *in vitro*, and that is capable of ubiquitylating the HIF-1a transcription factor as occurs in mammals [44], [45], [46]. Our data indicate that any potential redundancy between Cul2 and Cul5 in *Drosophila* must occur by utilizing different Roc proteins, as Roc1a is not part of a Cul5 complex, and Roc2 is not part of the Cul2 complex. Whether redundancy exists or not, that the Cul5-Roc2 complex has been evolutionarily conserved suggests that it plays an important role in many organisms.

## Materials and Methods

### Cell Culture and Transfection

S2 cells were maintained in Schneider's medium supplemented with 10% FBS and 100 U/ml penicillin and 100 ug/ml streptomycin. Cells were transfected using Effectene according to the manufacturer's instructions (Qiagen), and protein lysates were obtained 48 hours later. All transfected DNA's were cloned into the pCaSpeR-4 vector.

### Cloning

The RING swap constructs were made by using the CAICR protein sequence that is common to all *Drosophila* Roc proteins (see Fig. 1B) as a region of overlap for primer design. Chimeras were expressed with either a *Roc1a*- or *Roc1b*-grf (genomic rescue fragment). The *Roc1a*-grf was previously described [24] and contains 980 bp upstream of the Start codon and 620 bp downstream of the stop codon. A FLAG- or V5- tag was inserted in frame immediately downstream of the initiating methionine. The *Roc1b*-grf, containing 840 bp upstream from the Start codon and 330 bp downstream from the Stop codon, was also previously described [25], and here we inserted an in frame V5 tag downstream of the Start codon.

### Creation of GST-fusion Proteins

Using the pCaSpeR-4 Roc constructs as template [25], PCR products were made using primers that added EcoR1 sites on either side, and the product was then cloned into pGEX-1 (GE Healthcare) and confirmed by sequencing. Primers used are as follows. Roc1a 5′Eco: 5′-CAGAGGAATTCGAAGTCGACGAGGATGGATAC-3′. Roc1a 3′Eco: 5′-CAGAGGAATTCTTAGTGGCCGTACTTC-3′. Roc1b 5′Eco: 5′-TCATTAGAATTCGCCGAGGAGATAGAGGTTG-3′. Roc1b 3′Eco: 5′-CAGAGGAATTCACCGGTCTAGCGGCCGTACTTC-3′. Roc2 5′Eco: 5′-TGACAGGAATTCGCTGATGATCCAGAAAACTC-3′. Roc2 3′Eco: 5′-CAGAGGAATTCACCGGTTTATTTTCCCATGCG-3′.

Protocol used for isolation of GST fusion proteins was previously described [26]. Constructs were transformed into BL21DE3 bacteria and induced by adding IPTG to 0.4 mM. GST-Roc proteins were purified using Glutathione-Sepharose 4B beads (GE Healthcare). Protein concentration was determined by Coomassie stain using BSA standards.

### Ubiquitin Ligase Assay

Ligase assays were performed essentially as described [24] for 45 minutes at 37°C with the following components: 50 mM Tris-HCl pH 7.5, 5 mM MgCl_2_, 2 mM NaF, 0.6 mM DTT, 2 mM ATP, 10 nM Okadaic Acid, 40 ng rabbit Ube1 (Boston Biochem), 300 ng UbcH5, 12 µg bovine Ub (Sigma), and 250 ng GST-Roc. Samples were run by SDS-PAGE on a 12% gel followed by Western blotting for Ubiquitin.

### RT-PCR Analysis

RNA was extracted from embryos using TRIzol (Sigma-Aldrich) according to the manufacturer's instructions. RT-PCR was previously described [25].

Primer sequences used for RT-PCR are as follows. CG8234: 5′-CACCCATGTGTCCTTCTCCGT-3′ and 5′-TGACCACGGTTCACAAACCAG-3′. CG30035: 5′-GAGAACATCCGTCATGCGGTG-3′ and 5′-GAGCAGGATGCCTATGTTACC-3′. Cul5: 5′-CACAAAGTTCATTTGACGAGGCG-3′ and 5′-TGTGGCCAGGCGGAGATTCTC-3′. Roc2: 5′-CAGAGACCGGTATGGCTGATGATCCAGAA-3′ and 5′-CCCATGCGCTGAATGGACCA-3′.

### Immunoprecipitations and Western Blotting

For embryo lysates, overnight egg collections (0–16 hrs) were dechorionated for 3 minutes in 50% bleach and dounce homogenized with 10 volumes of NP-40 lysis buffer (50 mM Tris pH 8.3, 150 mM NaCl, 0.5% NP-40, 1 ng/ml leupeptin, 0.5 ng/ml pepstatinA, 1 mM PMSF). Lysates were centrifuged at 14,000 rpm for 10 min at 4°C and the supernatant was collected. For immunoprecipitations, 25 µl protein-A beads were washed 3× with 1 ml NP-40 lysis buffer, and then pre-incubated with antibody for 2 h before adding to 1 ml of lysate (1 mg/ml protein). Immunoprecipitations were performed overnight at 4°C.

### Antibodies

The following antibodies were used: rabbit anti-Cul1 (Zymed), rabbit anti-Cul2 (gift of Dr. Yue Xiong, UNC), guinea pig anti-Cul3 (gift of Dr. Jim Skeath, Washington University), rabbit anti-Cul4 [27], affinity purified rabbit anti-Cul5, guinea pig anti-Roc2 whole serum, mouse anti-FLAG (Invitrogen), mouse anti-V5 (Invitrogen), and Rabbit anti-Ub (Covance). Anti-peptide antibodies against Roc2 and Cul5 were generated using synthetic peptides (Invitrogen) coupled to KLH (Pierce). The peptide sequences are CADDPENSVDRPTDD (Roc2) and CKRDRDIFEEVWPDK (Cul5). Injections and serum withdrawal were performed at Pocono Rabbit Farm & Laboratory, Inc. High titer bleeds of anti-Cul5 were purified with the same peptide using the Sulfolink Kit (Pierce).

### Stocks and Genetics

The *Roc1a^G1^* and *Roc1b^dc3^* alleles have been described previously [24], [25]. The *Roc2^KG07982^*, *Roc2^pBacf00911^*, and *Cul5^EY21463^* alleles were obtained from the Bloomington stock center. To test for rescue of *Roc1a* lethality, *Roc1a^G1^*, *sn*, *FRT/FM7*, *Act-GFP* females were mated to males that expressed a specific transgene (V5-Roc1a or V5-ANBR) under control of the *Roc1a* promoter. Rescue was scored by the presence of *sn* males in the progeny. To test for rescue of *Roc1b* male sterility, males of the genotype *Roc1b^dc3^/TM3*, *Sb* and containing a transgene insertion on the second chromosome (V5-Roc1b, V5-ANBR, or V5-BNAR) were mated to *Roc1b^dc3^/TM3*, *Sb* females. *w^+^* (indicating the presence of the transgene), *Sb^+^*, *Roc1b^dc3^/Roc1b^dc3^* male progeny were then mated with *w^1118^* virgin females to assay for rescue of sterility. Five batches of 100 eggs from this cross were transferred onto individual grape juice plates, and the numbers of hatched eggs quantified 36 h later.
